# The Statistical Fragility of Functional Outcomes for Arthroscopic Rotator Cuff Repair With and Without Acromioplasty: A Systematic Review and Meta-analysis

**DOI:** 10.1177/03635465241302797

**Published:** 2025-01-21

**Authors:** David S. Clark, Benjamin C. Tingey, Jeffrey L. Shi, Jeremy S. Somerson

**Affiliations:** † John Sealy School of Medicine, University of Texas Medical Branch, Galveston, Texas, USA; ‡ Parkinson School of Health Sciences and Public Health, Loyola University Chicago, Maywood, Illinois, USA; § Department of Orthopaedic Surgery and Rehabilitation, University of Texas Medical Branch, Galveston, Texas, USA; Investigation performed at the University of Texas Medical Branch, Galveston, Texas, USA

**Keywords:** acromioplasty, arthroscopic surgery, rotator cuff, statistical analysis, statistics

## Abstract

**Background:**

Views surrounding acromioplasty at the time of arthroscopic rotator cuff repair (RCR) have shifted dramatically over time. In recent years, various studies have argued against acromioplasty, citing equivocal functional outcomes after arthroscopic RCR with or without acromioplasty.

**Purpose:**

To assess the statistical fragility of functional outcomes after arthroscopic RCR with and without acromioplasty using the reverse continuous fragility index (RCFI).

**Study Design:**

Systematic review and meta-analysis; Level of evidence, 3.

**Methods:**

A systematic review and meta-analysis was performed including all randomized controlled trials through February 5, 2024 investigating arthroscopic RCR with and without acromioplasty. The RCFI, defined as the number of qualifying data points required to be moved from the lower mean group to the higher mean group to alter the significance, was calculated for the Welch *t* test, Student *t* test, and Wilcoxon rank-sum test under various data assumptions. The reverse continuous fragility quotient (RCFQ) was determined by dividing the RCFI by the sample size.

**Results:**

A total of 6 clinical trials consisting of 609 patients with functional outcome scores were analyzed. Using the Welch *t* test, the median RCFI across all study outcomes was 20 (interquartile range [IQR], 17-24). For the Student *t* test, the median RCFI across all study outcomes was 14 (IQR, 13-19), with a median RCFQ of 0.18 (IQR, 0.15-0.20). For the Wilcoxon rank-sum test, the median RCFI was 14 (IQR, 13-17), with a median RCFQ of 0.17 (IQR, 0.13-0.19). While using the Welch *t* test, 64% of study outcomes had an RCFI greater than the loss to follow-up (LTFU). When using the other tests, 32% of study outcomes had an RCFI greater than the LTFU.

**Conclusion:**

The fragility of these studies was largely dependent on the statistical test used to analyze the results. The Wilcoxon rank-sum test and Student *t* test appeared to be most appropriate to find differences in treatment arms. When using these tests, we found the results to be fragile. This, in combination with a small number of studies and the LTFU close to or exceeding 20%, indicates an overall lack of strong evidence to support previously accepted conclusions.

The appropriate treatment method of rotator cuff disease remains controversial.^5^ In 1972, Neer^25^ published his findings advocating extrinsic mechanical impingement as the primary driver of rotator cuff disease. He proposed acromioplasty, removal of the anterior edge and inferior surface of the acromion and release of the coracoacromial ligament, to relieve extrinsic wear of the supraspinatus tendon. Since then, acromioplasty has become a mainstay in the treatment of rotator cuff disease, with its substantially increased use into the 1990s and mid-2000s.^22,34^ However, prospective randomized trials and basic science studies have raised uncertainty about the need for acromioplasty.^8,19,28^

The conclusions of these studies rely on the *P* value, which is calculated to determine whether the results are statistically significant.^10^ While valuable, the *P* value is not without its limitations, as noted in a statement by the American Statistical Association.^36^ More nuanced measures are needed to give full context and understanding to the results of a study.^3,4,32,33^ The fragility index (FI), first proposed by Feinstein^16^ in 1990, is one such tool providing clinicians with a more complete view of study results. The FI is defined as the number of event outcomes required to be altered to change the results from significant to insignificant.^14^ This is done by changing outcomes stepwise in one study arm until the *P* value exceeds the .05 cutoff.^17^ This process may be conducted in the opposite direction to calculate the reverse fragility index (RFI) to evaluate the fragility of nonsignificant results.^29^ These results can be standardized for differing sample sizes by calculating the fragility quotient (FQ) or reverse fragility quotient (RFQ), which is the FI or RFI divided by the total sample size.^33^ This indicates the percentage of the study population on which the study results rely. A larger FQ/RFQ indicates a more robust study requiring a higher percentage of the study population to be changed to alter significance. Additionally, these tools can be analyzed in consideration with a study’s loss to follow-up (LTFU). When the LTFU exceeds the FI/RFI, studies should be viewed with greater skepticism, as the unknown outcomes could alter the results.^14^

One key limitation of the FI/RFI is that it can only be applied to dichotomous outcomes.^35^ Caldwell et al^7^ recently introduced an alternative method to compute the continuous fragility index (CFI) to assess continuous outcomes, such as outcome scores or pain levels. We sought to apply this method with modifications to compute the reverse CFI (RCFI), which is the number of qualifying data points needed to be moved to achieve statistical significance. Our method will allow for fragility analysis of patient-reported functional outcomes, which have thus far been shown to be equivocal, for those undergoing arthroscopic rotator cuff repair (RCR) with or without acromioplasty.

## Methods

There were 2 electronic databases (PubMed/MEDLINE, Scopus) searched to identify articles on arthroscopic RCR with and without acromioplasty using the following search terms: “(acromioplasty OR subacromial decompression OR acromial resection OR acromion resection OR subacromial bursectomy) AND (rotator cuff)” through February 5, 2024.

Studies were eligible for inclusion if they met the following criteria: randomized controlled trials (RCTs) or controlled clinical trials; at least 12 months of follow-up; studies published as full articles; comparison of arthroscopic RCR with or without acromioplasty; and at least 1 reported functional outcome score for the Western Ontario Rotator Cuff Index (WORC), American Shoulder and Elbow Surgeons (ASES) score, University of California, Los Angeles (UCLA), or Constant score with the mean, standard deviation (SD), and sample size (by treatment arm) included.

**Figure 1.**
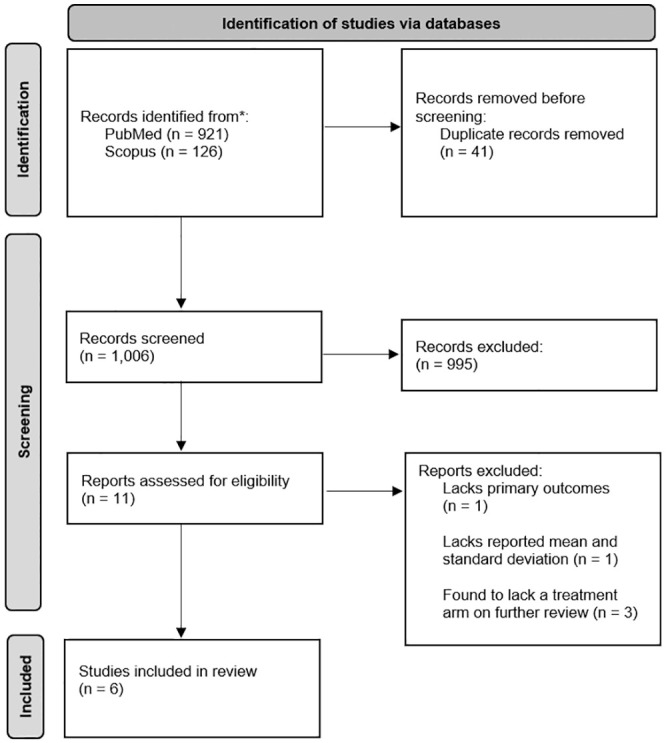
PRISMA (Preferred Reporting Items for Systematic Reviews and Meta-Analyses) diagram on article selection for this systematic review.

Studies were deemed ineligible for inclusion if the following were found: undefined sample size and control source; animal experiments; no reported mean for functional outcome scores; nonrandomized trials, biomechanical studies, case reports, and observational studies; nonoriginal studies; and undefined groups. The Preferred Reporting Items for Systematic Reviews and Meta-Analyses (PRISMA) guidelines were used in this review (Figure 1).

### Data Extraction and Outcomes

The reviewers extracted data from studies meeting the specified criteria, including the authors, year of publication, journal, sample size for each arm of the trial, length of follow-up, LTFU, and functional outcome scores according to the Likert scale (WORC [0-100], ASES [0-30], UCLA [0-35], Constant [0-100]).

### Statistical Analysis

The RCFI was calculated for all included study outcomes using the methods described by Caldwell et al.^7^ With adaptations made, we created a code to analyze the number of qualifying data points needed to be changed to achieve statistical significance at the specified *P* value (see Appendix 1, available in the online version of this article). Because raw data were not available from the studies, summary statistics (sample size, outcome mean and SD) were used to create a simulated candidate dataset for each treatment arm. After a candidate dataset was created, it was passed through a rejection algorithm and accepted if the mean and SD were within a specified tolerance of the input values. If the dataset was rejected, a new candidate dataset was simulated, and the process was repeated. When acceptable datasets for each arm were produced, the RCFI was determined by the following process: the group with the smaller mean was identified, and the data point just greater than the mean in that group was moved to the group with the larger mean. The process was repeated until the *P* value, calculated from the Welch *t* test, reached a level of statistically significant difference between the 2 groups. The final RCFI was reported as the number of qualifying data points moved. To further evaluate the RCFI, given the potential differences arising from different distributional assumptions, significance testing was repeated using the Student *t* test and the nonparametric Wilcoxon rank-sum test with the same simulated datasets.

Further, to account for possible bias in results stemming from the simulated datasets, the entire process was repeated an iterative number of times, and the RCFI was averaged across iterations. To determine the extent of agreement of RCFI values between different iterations of simulated datasets, the intraclass correlation coefficient (ICC) was calculated, with an ICC <0.05 indicating poor agreement and an ICC ≥0.90 indicating excellent agreement. The ICC was tested on RCFI calculations from various simulated dataset iterations (n = 1, 5, 10, 50, 100). The ICC was 0.961 (95% confidence interval, 0.907-0.988), indicating excellent agreement between the different iterations. Based on these findings, 10 simulated datasets over which the RCFI was averaged were used for each study outcome.

The reverse continuous fragility quotient (RCFQ) was calculated by dividing the RCFI by the total study sample size to determine the proportion of qualifying data points moved relative to the study’s sample size. We report the median (interquartile range [IQR]) RCFI/RCFQ across the studies overall. Additionally, we report each study’s individual RCFI/RCFQ along with the LTFU to compare to RCFI/RCFQ estimates. All hypothesis tests were 2-sided, with the default significance threshold (alpha) set to .05 for all analyses. As supplemental analysis, RCFI/RCFQ calculations were repeated while adjusting the significance threshold from .05 to .01 and .005. The median (IQR) RCFI/RCFQ values were again presented across the studies overall. All analyses were performed with R (Version 4.3.1; R Foundation for Statistical Computing).

## Results

Of the 1047 studies initially screened, 6 RCTs were included for analysis after screening and fulfilling eligibility criteria. Summary characteristics of each study are presented in Table 1. A total of 609 patients with functional outcome scores were analyzed. Enrollment sizes ranged from 80 to 150. Follow-up times ranged from 12 months to 11.5 years, with the LTFU ranging from 0 to 30. Each study analyzed concluded no difference in functional outcomes for RCR with and without acromioplasty.^1,18,23,24,30,37^ One study, Woodmass et al,^37^ is a long-term follow-up of a previous study also included in our analysis.

**Table 1:** Study Characteristics^*a*^

First Author (Year)	No. of RCR Cases (With:Without Acromioplasty)	Mean Age, With:Without Acromioplasty, y	Minimum Follow-up, mo	Mean Follow-up	No. of Patients Lost to Follow-up	Total No. of Enrolled Patients
Woodmass^37^ (2022)	56 (25:31)	56.2:58.5		11.2-11.5 y	30	86
Milano^24^ (2007)	71 (34:37)	61.0:59.7	24	24 mo	9	80
Gartsman^18^ (2004)	93 (47:46)	59.3:60.0	12	15.6 mo	0^*b*^	93
Shin^30^ (2012)	120 (60:60)	57.8:55.8	24	35 mo	30	150
Abrams^1^ (2014)	95 (52:43)	59.6:58.0	24		19	114
MacDonald^23^ (2011)	68 (32:36)	56.8	24		18	86

a RCR, rotator cuff repair.

b No loss to follow-up was reported in this study, and the number was assumed to be 0.

Overall study median values for the RCFI and RCFQ are reported in Table 2 for the 3 different statistical tests. Using the Welch *t* test, the median RCFI across all study outcomes was 20 (IQR, 17-24), indicating that 20 qualifying data points were required to be moved to change test results from nonsignificant to significant. The median RCFQ was 0.24 (IQR, 0.21-0.26), indicating that the nonsignificance of results was contingent on 24 qualifying data points per 100 participants. Values are also reported from the other statistical tests, of which both were similarly lower than those of the Welch *t* test. For the Student *t* test, the median RCFI across all study outcomes was 14 (IQR, 13-19), with a median RCFQ of 0.18 (IQR, 0.15-0.20). For the Wilcoxon rank-sum test, the median RCFI was 14 (IQR, 13-17), with a median RCFQ of 0.17 (IQR, 0.13-0.19).

**Table 2:** RCFI and RCFQ Across All Outcome Scores (α = .05)^*a*^

	RCFI	RCFQ
Welch *t* test	20 (17-24)	0.24 (0.21-0.26)
Wilcoxon rank-sum test	14 (13-17)	0.17 (0.13-0.19)
Student *t* test	14 (13-19)	0.18 (0.15-0.20)

a Data are presented as median (interquartile range). Each individual outcome score was averaged over 10 iterated simulated datasets, and the median was taken of all similarly calculated outcomes. RCFI, reverse continuous fragility index; RCFQ, reverse continuous fragility quotient.

RCFI and RCFQ values for each individual study for each statistical test are displayed in Table 3. For the Welch *t* test, the LTFU was greater than the RCFI in 36% (4/11) of studies. However, for the Student *t* test and Wilcoxon rank-sum test, the LTFU was greater than the RCFI in 64% (7/11) and 73% (8/11) of studies, respectively.

**Table 3:** RCFI and RCFQ for Individual Outcome Scores Compared With LTFU (α = .05)^*a*^

	Welch *t* Test		Wilcoxon Rank-Sum Test		Student *t* Test		
	RCFI	RCFQ	RCFI	RCFQ	RCFI	RCFQ	LTFU
WORC							
Woodmass^37^	17 ± 4.58	0.30	13 ± 2.87	0.23	13 ± 2.42	0.23	30
MacDonald^23^	18 ± 3.20	0.26	13 ± 3.58	0.19	13 ± 1.65	0.19	18
ASES							
Gartsman^18^	22 ± 4.65	0.24	15 ± 3.95	0.16	17 ± 2.97	0.18	0
Shin^30^	21 ± 3.92	0.18	16 ± 3.63	0.13	19 ± 3.27	0.16	30
Abrams^1^	24 ± 4.95	0.25	17 ± 4.20	0.18	19 ± 2.68	0.20	19
MacDonald^23^	20 ± 7.84	0.29	12 ± 3.09	0.18	13 ± 3.86	0.19	18
UCLA							
Shin^30^	14 ± 3.57	0.12	13 ± 3.50	0.11	12 ± 2.00	0.10	30
Abrams^1^	25 ± 4.22	0.26	19 ± 3.23	0.20	20 ± 1.84	0.21	19
Constant							
Milano^24^	15 ± 3.55	0.21	9 ± 2.91	0.13	11 ± 1.17	0.15	9
Shin^30^	28 ± 4.60	0.23	20 ± 2.92	0.17	22 ± 2.13	0.18	30
Abrams^1^	20 ± 3.71	0.21	14 ± 4.38	0.15	14 ± 1.97	0.15	19

a Data are presented as mean ± SD unless otherwise indicated. Each individual outcome score was averaged over 10 iterated simulated datasets. ASES, American Shoulder and Elbow Surgeons; LTFU, loss to follow-up; RCFQ, reverse continuous fragility quotient; UCLA, University of California, Los Angeles; WORC, Western Ontario Rotator Cuff Index.

Additional analysis results are displayed in Table 4, with overall study median values for the RCFI and RCFQ being reported again for all 3 different statistical tests while also presenting values calculated at more stringent significance thresholds at α = .01 and α = .005. In all cases, median RCFI/RCFQ values increased when decreasing the significance threshold, but values remained consistently lower when using the Student *t* test or Wilcoxon rank-sum test compared with the Welch *t* test.

**Table 4:** RCFI and RCFQ Across All Outcome Scores Stratified by Significance^*a*^

	α = .05		α = .01		α = .005	
	RCFI	RCFQ	RCFI	RCFQ	RCFI	RCFQ
Welch *t* test	20 (17-24)	0.24 (0.21-0.26)	28 (25-33)	0.35 (0.30-0.37)	33 (27-37)	0.38 (0.35-0.40)
Wilcoxon rank-sum test	14 (13-17)	0.17 (0.13-0.19)	19 (15-24)	0.22 (0.20-0.24)	21 (18-25)	0.24 (0.21-0.26)
Student *t* test	14 (13-19)	0.18 (0.15-0.20)	20 (16-25)	0.23 (0.20-0.26)	23 (18-27)	0.25 (0.23-0.27)

a Data are presented as median (interquartile range). Each individual outcome score was averaged over 10 iterated simulated datasets. RCFI, reverse continuous fragility index; RCFQ, reverse continuous fragility quotient.

## Discussion

In our evaluation of the statistical fragility of RCTs comparing the functional outcomes of arthroscopic RCR with and without acromioplasty using the most appropriate statistical tests (Student *t* test and Wilcoxon rank-sum test), the results were fragile. Few RCFI values were greater than the LTFU (32%), calling previous conclusions about the equivalence of arthroscopic RCR with or without acromioplasty into question.

The Welch *t* test is claimed to be a more readily applied test because of its less restrictive assumptions.^27^ This aspect, among others, is why this particular test is used as a default statistical test in situations in which the underlying distributional properties of study data are unknown.^11,12,26^ However, while the Welch *t* test can be widely applied in various scenarios, such as the true equality of variance, it is known to be a conservative test. This means that it reveals systematically higher *P* values than should be expected,^15^ such that in the case of fragility, it would make results appear more robust than they actually are.

Previous research has shown that under different scenarios, the Student *t* test, also assuming equal variance, achieves higher power than the Welch *t* test.^31^ Because of this, the Student *t* test may be better suited to assess the true fragility of study results. Additionally, without the assumption of a normal distribution, the Welch *t* test has been shown to lead to systematically biased results and extremely small *P* values.^9^ This again may inflate the perceived robustness of study results. Assuming a normal distribution in many circumstances can be relaxed with larger sample sizes.^21^ However, in the case of small datasets, this assumption becomes more important. Şimşek^31^ found the Wilcoxon rank-sum test to be superior to the Welch *t* test and Student *t* test when using Likert-type data, particularly in scenarios of small samples and unequal group sizes. Noting the findings of such literature and coupled with those of this study, we argue against the use of the Welch *t* test as a “one-size-fits-all” for any continuous outcome when determining fragility. The practice of employing the same “go-to” statistical test for study data without any inspection of distributional properties could possibly result in misleading findings.

We believe that greater justification should be given to the fragility of results found using the Student *t* test and the Wilcoxon rank-sum test, given that the Wilcoxon rank-sum test results were comparable with those of the Student *t* test for the following reasons: (1) study sample sizes were small; (2) arm samples were not largely different from each other; (3) arm SD values were within less than a 1.5 ratio, indicating a descriptive equality of variance assumption met^6^ (see Appendix Table A1, available online); and (4) outcome types mirrored similar data types to those reported in Şimşek.^31^ Additionally, several^23,30,37^ of the studies included reported LTFU values at or greater than 20% of the study population. This level of LTFU already calls into question the level of evidence presented by these studies.

One study^18^ did not report LTFU. The decision was made to assume 0 LTFU to produce a conservative estimate of the fragility of this topic. When this study is removed from consideration, the RCFI greater than the LTFU drops to 50% when using the Welch *t* test and 25% when using the Student *t* test and Wilcoxon rank-sum test. Previous research evaluating the CFI, using the Welch *t* test only, of studies evaluating treatment approaches for anterior shoulder instability found that 61.4% of those studies had a CFI greater than the LTFU, which is comparatively more robust than the results of the present study.^2^ With greater fragility in results yielding RCFI values largely less than the LTFU, along with concerns over the percentage of LTFU and reporting, the conclusion of previous meta-analyses claiming that arthroscopic RCR with or without acromioplasty produces equivocal functional outcomes is thus questioned.^8,20,28^

Our study is limited by the number of qualifying studies producing only a small amount of data to analyze; however, this further supports the conclusion of a lack of robustness in findings and more work needing to be done to confirm the results. In this analysis, we were also limited to the reported summary statistics of each study to produce simulated datasets and cannot make use of original raw data. We relied on the authors reporting their statistics and their LTFU to obtain the most accurate estimate of the fragility of the study results. Although we employed measures to produce averaged results across several iterative sets that met tolerance criteria and agreement metrics (ICC = 0.96), our results are still only contingent on such artificial sets. To compensate, we applied 3 statistical tests uniformly across all studies, but the best approach would be to apply the test that is most appropriate to the specific data. Thus, although our analyses suggest the results to be more fragile, this could only be more definitively understood by investigating the original data on a study-by-study basis. Additionally, one of the primary limitations of the FI is the lack of a standard cutoff delineating robust versus fragile findings.^14^ There is no standard to ascertain whether a certain study is deemed robust based on the FI alone; it must be taken into consideration along with the LTFU to analyze the study in its entire context. Despite these limitations, we applied rigorous simulation techniques to acquire representative data and presented the data under various assumptions to provide a comprehensive analysis of fragility.

## Conclusion

The current literature analyzing the functional outcomes of arthroscopic RCR with and without acromioplasty has been interpreted to show equivocal outcomes. Our analysis shows that the fragility of these study results was largely dependent on the statistical test used to analyze the results. Using tests that were most appropriate to find differences in the treatment arms (Wilcoxon rank-sum test and Student *t* test), we found the results to be fragile. This, in combination with a small number of studies and the LTFU close to or exceeding 20%, indicates an overall lack of strong evidence to support previously accepted conclusions.^13^ Further prospective studies with robust statistical power and a limited LTFU are important to support or reject the importance of acromioplasty in conjunction with RCR. The method described in this work also provides a tool for other researchers to assess the reverse fragility of continuous outcomes in other areas of orthopaedic surgery.

## Supplemental Material

sj-pdf-1-ajs-10.1177_03635465241302797 – Supplemental material for The Statistical Fragility of Functional Outcomes for Arthroscopic Rotator Cuff Repair With and Without Acromioplasty: A Systematic Review and Meta-analysis Supplemental material, sj-pdf-1-ajs-10.1177_03635465241302797 for The Statistical Fragility of Functional Outcomes for Arthroscopic Rotator Cuff Repair With and Without Acromioplasty: A Systematic Review and Meta-analysis by David S. Clark, Benjamin C. Tingey, Jeffrey L. Shi and Jeremy S. Somerson in The American Journal of Sports Medicine
