# Supplementary material for: The Statistical Fragility of Functional Outcomes for Arthroscopic Rotator Cuff Repair With and Without Acromioplasty: A Systematic Review and Meta-analysis
Source: Am J Sports Med. 2025 Jan 21;53(10):2483–8. doi: 10.1177/03635465241302797 (PMC12311245; doi:10.1177/03635465241302797)
Supplement: sj-pdf-1-ajs-10.1177_03635465241302797 – Supplemental material for The Statistical Fragility of Functional Outcomes for Arthroscopic Rotator Cuff Repair With and Without Acromioplasty: A Systematic Review and Meta-analysis [file sj-pdf-1-ajs-10.1177_03635465241302797.pdf]

The Statistical Fragility of Functional Outcomes for Arthroscopic Rotator Cuff Repair With and Without Acromioplasty

APPENDIX 1

```
#ContFrag.R - An R script for Calculating Fragility Index for Continuous
#Variables
#Author: Jon-Michael E. Caldwell
#Last Revision: 5/23/2020
#Functions:
# contFrag(): Function for batch processing multiple sets of inputs (studies)
#from an excel file, outputs saved to output.csv in same folder. Paths are
#hardcoded.
# mainLoop(): Run algorithm for given set of variables. Can be called by
#contFrag() or directly with supplied arguments (see function definition for
#                                   arguments).
# calcFragility(): Loop to call to tradeMeans(), test significance, and
#increment a counter until significance is lost
# tradeMeans(): sort and find datapoint closest to mean in one dataset, trade
#to other dataset
# createStudy(): set up new datasets and display summary stats
# makeData(): create simulated Dataset with given parameters
# resetVars(): clear global variables
#Usage:
# For batch processing:
# -Run script to load functions
# -Console command: contFrag() //will need to have studies.xlsx at hardcoded
```

```
#path
#Change Log
#v3 - added round() to report of AverageFI to only report whole numbers. This
#makes more sense
# - fixed path errors in file names
# - Added tryCatch() to calcFragility() to deal with "infinite" FI for studies
#that do not achieve insignificance.
# This change will set the FI for that run equal to the N being shifted
# - Added comments to trademeans() and removed trademeansMax() function
#(depricated)


#####################################################################################
########## RUN function shells

averagefi_WELCH = NULL
averagefi_EQVAR = NULL
averagefi_RANKSUM = NULL
outputs= NULL
meanChanges = NULL
nChanges = NULL


resetVars <- function() {
  controlData <<- NULL
  testData <<- NULL
  ttest <<- NULL
  fi_WELCH <<- 0
  fi_EQVAR <<- 0
```

```
  fi_RANKSUM <<- 0
}

makeData <- function (target_mean, target_std, n, attempts, sensitivity) {
  i = 0
  newScore = 9999
  oldScore = 9999
  theData = NULL
  newData = NULL
  while(oldScore > sensitivity) {
    newData <- rnorm(n, mean = target_mean, sd = target_std) #make a new dataset
    newScore = abs(mean(newData)-target_mean) + abs(sd(newData)-target_std)
    if (newScore <= oldScore) { #test to see if new data is better fit than old
      oldScore <- newScore
      theData <- newData
    }
    i = i + 1
    if (i > attempts) { #break if taking too long .. maybe take this out?
      print("Tried it all of the times and no dice, we returned the best
option")
      break;
    }
  }
  return(theData)
}


createStudy <- function (controlMean, controlSD, controlN, testMean, testSD,
                         testN) {
```

```
  #generate data sets
  controlData <<- makeData(controlMean, controlSD, controlN, 500000, 0.01)
  testData <<- makeData(testMean, testSD, testN, 500000, 0.01)
  print("Control Data:")
  print(summary(controlData))
  print("Test Data:")
  print(summary(testData))

  controlData_WELCH <<- controlData
  testData_WELCH <<- testData

  controlData_EQVAR <<- controlData
  testData_EQVAR <<- testData

  controlData_RANKSUM <<- controlData
  testData_RANKSUM <<- testData
}


tradeMeans_WELCH <- function (x,y) {
  x <- sort(x)
  y <- sort(y)
  xmean <- mean(x)
  ymean <- mean(y)
  print(xmean)
  print(ymean)
  ##########################################################################################
  ######## RCFI, edit
  ### if y mean is greater, move closest x point to x mean (but still greater than x mean) to y
```

```
#############################################################################################
if (xmean < ymean) { #run this block if control mean < test mean
  if (x[which.min(abs(x-mean(x)))] > xmean) { #pick the point closest to but > mean
    index = which.min(abs(x-mean(x)))
  }
  else{
    index = which.min(abs(x-mean(x))) + 1
  }
  freeagent <- x[index] #copy the patient to move
  x <- x[-index] #remove freeagent from x
  y <- append(y, freeagent) #add freeagent to y
  cat("Moving a patient from control to test group with value: ", freeagent)
  print(freeagent)
}
else{ #run this block if control mean > test mean
  if (y[which.min(abs(y-mean(y)))] > ymean) {
    index = which.min(abs(y-mean(y)))
  }
  else{
    index = which.min(abs(y-mean(y))) + 1
  }
  freeagent <- y[index] #copy the patient to move
  y <- y[-index] #remove freeagent from y
  x <- append(x, freeagent) #add freeagent to x
  cat("Moving a patient from test to control group with value: ",
      freeagent,"\n")
}
controlData_WELCH <<- x
testData_WELCH <<- y
```

```
}

tradeMeans_EQVAR <- function (x,y) {
 x <- sort(x)
 y <- sort(y)
 xmean <- mean(x)
 ymean <- mean(y)
 print(xmean)
 print(ymean)
 ###############################################################################
 ######## RCFI, edit
 ### if y mean is greater, move closest x point to x mean (but still greater than x mean) to y
 ###############################################################################
 if (xmean < ymean) { #run this block if control mean < test mean
  if (x[which.min(abs(x-mean(x)))] > xmean) { #pick the point closest to but > mean
   index = which.min(abs(x-mean(x)))
  }
  else{
   index = which.min(abs(x-mean(x))) + 1
  }
  freeagent <- x[index] #copy the patient to move
  x <- x[-index] #remove freeagent from x
  y <- append(y, freeagent) #add freeagent to y
  cat("Moving a patient from control to test group with value: ", freeagent)
  print(freeagent)
 }
 else{ #run this block if control mean > test mean
  if (y[which.min(abs(y-mean(y)))] > ymean) {
   index = which.min(abs(y-mean(y)))
```

```
    }
    else{
      index = which.min(abs(y-mean(y))) + 1
    }
    freeagent <- y[index] #copy the patient to move
    y <- y[-index] #remove freeagent from y
    x <- append(x, freeagent) #add freeagent to x
    cat("Moving a patient from test to control group with value: ",
        freeagent,"\n")
  }
  controlData_EQVAR <<- x
  testData_EQVAR <<- y
}

tradeMeans_RANKSUM <- function (x,y) {
  x <- sort(x)
  y <- sort(y)
  xmean <- mean(x)
  ymean <- mean(y)
  print(xmean)
  print(ymean)
  ##########################################################################################
  ######## RCFI, edit
  ### if y mean is greater, move closest x point to x mean (but still greater than x mean) to y
  ##########################################################################################
  if (xmean < ymean) { #run this block if control mean < test mean
    if (x[which.min(abs(x-mean(x)))] > xmean) { #pick the point closest to but > mean
      index = which.min(abs(x-mean(x)))
    }
```

```
    else{
      index = which.min(abs(x-mean(x))) + 1
    }
    freeagent <- x[index] #copy the patient to move
    x <- x[-index] #remove freeagent from x
    y <- append(y, freeagent) #add freeagent to y
    cat("Moving a patient from control to test group with value: ", freeagent)
    print(freeagent)
  }
  else{ #run this block if control mean > test mean
    if (y[which.min(abs(y-mean(y)))] > ymean) {
      index = which.min(abs(y-mean(y)))
    }
    else{
      index = which.min(abs(y-mean(y))) + 1
    }
    freeagent <- y[index] #copy the patient to move
    y <- y[-index] #remove freeagent from y
    x <- append(x, freeagent) #add freeagent to x
    cat("Moving a patient from test to control group with value: ",
        freeagent,"\n")
  }
  controlData_RANKSUM <<- x
  testData_RANKSUM <<- y
}


calcFragility_WELCH <- function (siglevel) {
    tryCatch ( {
```

```
    test <- t.test(controlData_WELCH, testData_WELCH, var.equal=FALSE) ############ designation for
Welch
   } ,
   error= function(e) {
    cat("*****ERROR: Infinite FI Detected***** Setting FI to N")
    fi_WELCH <<- fi_WELCH + 1
    test$p.value <<- 99
   }
   )
  currentp <- test$p.value #grab the current p-value
  cat("Current p-value: ", currentp, "\n")
  ####################################################################################
  ######## RCFI, edit
  ### if comparison p is GREATER than siglevel, continue algorithm
  ####################################################################################
  if (currentp > siglevel) { #if t-test is still sig, trade a patient
   tradeMeans_WELCH(controlData_WELCH, testData_WELCH)
   fi_WELCH <<- fi_WELCH + 1 #increment the FI
   return(0)
  }
  else{
   return(1)
  }
}

calcFragility_EQVAR <- function (siglevel) {
  tryCatch ( {
   test <- t.test(controlData_EQVAR, testData_EQVAR, var.equal = TRUE) ############ designation for
equal variances
```

```
 } ,
 error= function(e) {
  cat("*****ERROR: Infinite FI Detected***** Setting FI to N")
  fi_EQVAR <<- fi_EQVAR + 1
  test$p.value <<- 99
 }
 )
 currentp <- test$p.value #grab the current p-value
 cat("Current p-value: ", currentp, "\n")
 ##########################################################################################
 ######## RCFI, edit
 ### if comparison p is GREATER than siglevel, continue algorithm
 ##########################################################################################
 if (currentp > siglevel) { #if t-test is still sig, trade a patient
  tradeMeans_EQVAR(controlData_EQVAR, testData_EQVAR)
  fi_EQVAR <<- fi_EQVAR + 1 #increment the FI
  return(0)
 }
 else{
  return(1)
 }
}

calcFragility_RANKSUM <- function (siglevel) {
 tryCatch ( {
  test <- wilcox.test(controlData_RANKSUM, testData_RANKSUM) ############ designation for non-
parametric test
 } ,
 error= function(e) {
```

```
    cat("*****ERROR: Infinite FI Detected***** Setting FI to N")
    fi_RANKSUM <<- fi_RANKSUM + 1
    test$p.value <<- 99
  }
  )
  currentp <- test$p.value #grab the current p-value
  cat("Current p-value: ", currentp, "\n")
  ######################################################################################
  ######## RCFI, edit
  ### if comparison p is GREATER than siglevel, continue algorithm
  ######################################################################################
  if (currentp > siglevel) { #if t-test is still sig, trade a patient
    tradeMeans_RANKSUM(controlData_RANKSUM, testData_RANKSUM)
    fi_RANKSUM <<- fi_RANKSUM + 1 #increment the FI
    return(0)
  }
  else{
    return(1)
  }
}

mainLoop <- function(controlMean, controlSD, controlN, testMean, testSD,testN,
                     iterations, sensitivity,
                     siglevel) {
  a= 1:iterations
  averagefi_WELCH <<- NULL #reset the average FI at the beginning of a run
  averagefi_EQVAR <<- NULL
  averagefi_RANKSUM <<- NULL
  for (val in a) {
```

```
cat("Starting run", val, "of", iterations, "\n")
resetVars ()
createStudy(controlMean, controlSD, controlN, testMean, testSD, testN)
#origControl <- controlData
#origTest <- testData


done<- calcFragility_WELCH(siglevel)
while (done< 1) {
  done<- calcFragility_WELCH(siglevel)
}
cat("---Reverse Fragility Index (Welch's t-test): ", fi_WELCH, "---\n\n")
averagefi_WELCH <<- append(averagefi_WELCH, fi_WELCH)


done<- calcFragility_EQVAR(siglevel)
while (done< 1) {
  done<- calcFragility_EQVAR(siglevel)
}
cat("---Reverse Fragility Index (Equal variance t-test): ", fi_EQVAR, "---\n\n")
averagefi_EQVAR <<- append(averagefi_EQVAR, fi_EQVAR)


done<- calcFragility_RANKSUM(siglevel)
while (done< 1) {
  done<- calcFragility_RANKSUM(siglevel)
}
cat("---Reverse Fragility Index (Wilcoxon rank-sum test): ", fi_RANKSUM, "---\n\n")
averagefi_RANKSUM <<- append(averagefi_RANKSUM, fi_RANKSUM)
```

```
 }
 cat("\n***Average RFI (Welch's t-test) after ",length(a)," iterations is",
    round(mean(averagefi_WELCH))," +/- ",sd(averagefi_WELCH), "***")
 #return(round(mean(averagefi_WELCH))) #Round only the MEAN FI to minimize rounding  error

 cat("\n***Average RFI (Equal variance t-test) after ",length(a)," iterations is",
    round(mean(averagefi_EQVAR))," +/- ",sd(averagefi_EQVAR), "***")

 cat("\n***Average RFI (Wilcoxon rank-sum test) after ",length(a)," iterations is",
    round(mean(averagefi_RANKSUM))," +/- ",sd(averagefi_RANKSUM), "***")
}




#########################################################################################
########## FEED in study parameters and get RCFI
mainLoop(x, #control mean
        x, #control sd
        x, #control n

        x, #test mean
        x, #test sd
        x, #test n

        x, #number of simulated datasets (to average things over) (1,5,10,50,100)
        0.01, #tolerance criteria for building of simulated dataset
```

```
	0.05 #signifiance level
)
############################################################################################
```

Table A1. Summary Statistics for Included Studies

WORC

	Acromioplasty			Non-Acromioplasty		
**Author**	**Mean**	**SD**	**N**	**Mean**	**SD**	**N**
Woodmass JM	82.2	19.2	25	76.1	24.2	31
MacDonald P	87.5	15.3	32	80.7	21.3	36

UCLA

	Acromioplasty			Non-Acromioplasty		
**Author**	**Mean**	**SD**	**N**	**Mean**	**SD**	**N**
Shin SJ	33.4	3.3	60	32.3	3.5	60
Abrams GD	17.4	3.3	52	17.2	3.4	43

ASES

	Acromioplasty			Non-Acromioplasty		
**Author**	**Mean**	**SD**	**N**	**Mean**	**SD**	**N**
Gartsman GM	91.5	10.3	47	89.2	15.1	46
Shin SJ	90.7	13.1	60	87.5	12	60
Abrams GD	89	16.4	52	91.5	13.3	43
MacDonald P	90.5	13.4	32	85.6	19.1	36

CONSTANT

	Acromioplasty			Non-Acromioplasty		
**Author**	**Mean**	**SD**	**N**	**Mean**	**SD**	**N**
Milano G	103.6	17.5	34	96.1	20.9	37
Shin SJ	85	11.3	60	83.3	13	60
Abrams GD	78.7	11.1	52	75	15	43

Supplemental Table I. Mean, SD, N of functional outcome scores for each treatment arm in each included study.
